# Supplementary material for: An open-source framework for large-scale, flexible evaluation of biomedical text mining systems
Source: J Biomed Discov Collab. 2008 Jan 29;3:1. doi: 10.1186/1747-5333-3-1 (PMC2276192; doi:10.1186/1747-5333-3-1)
Supplement: Additional file 1 — Evaluation platform source code. The additional file contains the software evaluation framework discussed in this paper. The most current release can be downloaded from SourceForge.net [1]. See the accompanying README file for instructions on installation and use. Also included in the distribution is a collection of UIMA wrappers for some commonly used BioNLP tools and annotated corpora. [file 1747-5333-3-1-S1.zip › bionlp_uima_repository-1.0.1/docs/api/src-html/edu/uchsc/ccp/util/nlp/annotation/InvalidSpanException.html]

```
001    package edu.uchsc.ccp.util.nlp.annotation;
002    
003    public class InvalidSpanException extends Exception {
004    
005    
006        /**
007         * 
008         */
009        private static final long serialVersionUID = 6603176856454306034L;
010    
011        /**
012         * @param message
013         */
014        public InvalidSpanException(String message) {
015            super(message);
016        }
017    
018    
019    }
```
